# Potentiality, Limitations, and Consequences of Different Experimental Models to Improve Photodynamic Therapy for Cancer Treatment in Relation to Antiangiogenic Mechanism

**DOI:** 10.3390/cancers12082118

**Published:** 2020-07-30

**Authors:** Martin Majerník, Rastislav Jendželovský, Peter Fedoročko

**Affiliations:** Institute of Biology and Ecology, Faculty of Science, Pavol Jozef Šafárik University in Košice, Šrobárova 2, 041 54 Košice, Slovakia; martin.majernik@upjs.sk (M.M.); peter.fedorocko@upjs.sk (P.F.)

**Keywords:** tumor angiogenesis, photodynamic therapy, hypericin, photosensitizer, cell culture, spheroids, chorioallantoic membrane, in vivo

## Abstract

The relevance of experimentally gained information represents a long-term debating issue in the field of molecular biology research. The loss of original conditions in the in vitro environment affects various biological mechanisms and cellular interactions. Consequently, some biochemical mechanisms are lost or critically altered. Analyses in these modified conditions could, therefore, distort the relevancy of experimentally gained information. In some cases, the similarities with original conditions are so small that utilization of simpler in vitro models seems impossible, or could occur in a very limited way. To conclude, the study of more complex phenomena places higher demands on the complexity of the experimental model. The latest information highlights the fact that the tumor angiogenesis mechanism has very complex features. This complexity can be associated with a wide range of angiogenic factors expressed by a variety of malignant and non-malignant cells. Our article summarizes the results from various experimental models that were utilized to analyze a photodynamic therapy effect on tumor angiogenic mechanisms. Additionally, based on the latest information, we present the most important attributes and limitations of utilized experimental models. We also evaluate the essential problems associated with angiogenic mechanism induction after photodynamic therapy application.

## 1. Introduction

The era of modern photodynamic therapy (PDT) was begun by Lipson and Baldez in the 1960s, when fluorescence of neoplastic lesions after injection of hematoporphyrin (Hem) was observed [1]. PDT is considered a promising and very quickly developing area in the field of cancer research [2]. In the last fifty years, a lot of essential questions focused on PDT mechanisms, its utilization in treatment of malignant and non-malignant diseases, and potential limitations of this therapy have been answered [3,4,5,6]. However, the increasing number of novel findings makes us pose novel questions. Nowadays, the effect of photosensitizers (PS) alone or in a combination with PDT has been analyzed from different aspects [7,8,9,10,11,12,13,14]. In this context, the effect on angiogenesis represents one of the most studied issues (see Table 1, Table 2 and Table 3). One of the most preferred advantages of PDT is a higher selectivity for malignant cells, minimal or ideally no accumulation in non-malignant tissues, and reduced or no toxicity of the photosensitizer in dark conditions. These attributes of PDT are in contrast to the massively utilized chemotherapy and radiotherapy, mainly in relation to a strong targeting aspect of PDT and side effects minimization [2].

Despite a general effort to minimize side effects occurrence, there is evidence of its constant presence. The results of a huge amount of analyses showed that side effects evoked by PDT utilization belong to the major reasons not only for lowering therapeutic efficiency, but, moreover, they are associated with some pathological conditions’ development in PDT-affected areas. A wide range of analyses point to a tumor angiogenic mechanism induction as one of the most serious pathological conditions evoked specifically by PDT treatment (see Table 1). Moreover, the latest data emphasize angiogenic mechanism complexity and point to the fact that this mechanism directly depends on tumor environment presence and reciprocal interaction of malignant and non-malignant cells. However, there is much evidence that non-malignant cells affected by PDT may represent consequential angiogenesis promoting components after a treatment incidence [15,16,17,18,19,20].

Based on these facts, we assume that the targeting potential of PDT represents a fundamental condition for the extension of applied potential for a wide range of tumors of different histological origins. Simultaneously, the development of novel experimental models convenient for angiogenic research now represents a very interesting and examined area in the field of tumor angiogenesis research [14,21,22]. Moreover, an appreciable effort has been dedicated to a PDT study as a combined therapeutic approach with multiple antiangiogenic agents utilized [15,17,20,23,24,25,26,27,28,29]. Furthermore, a considerable effort has been focused on novel carriers, such as liposomes [30,31], dipolar solvents, e.g., N-methyl-pyrrolidone [32,33], polyether compounds, e.g., polyethylene glycol (PEG) [34], cyclodextrins [35], or nanoparticles [31]. We assumed that all these novel approaches could represent a potential solution for a PDT improvement in relation to tumor angiogenic mechanism inhibition.

## 2. Experimental Models and Their Potential, Limitations, and Relevancy Evaluation in Relation to PDT and Angiogenesis: Retrospective Summarization and Future Perspectives

**Figure 1:**
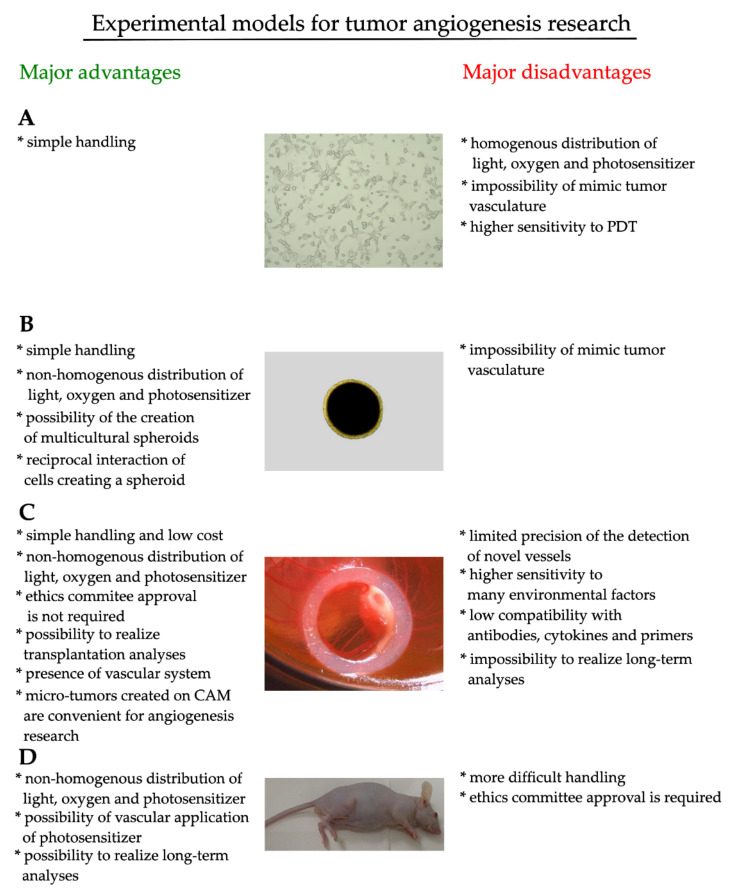
Major advantages and disadvantages of convenient experimental models for tumor angiogenesis research utilized. Picture (**A**) represents the 2D experimental model, (**B**) represents the 3D experimental model known as a spheroid, (**C**) represents the micro-tumor created on the quail (*Coturnix japonica*) avian embryo, and (**D**) represents the tumor-bearing mice.

Biological experiments focused on the analysis of a huge spectrum of molecular mechanisms are possible due to the use of experimental models, such as culture systems and animal models (see Figure 1) [69]. So far, it has been more than one hundred years since the first note of cell culture cultivation [70]. Since then, the cell cultivating protocol quality has improved extensively, and we can observe a persisting effort in the development of new protocols and cultivation techniques [14,71,72]. Now, established malignant and non-malignant experimental cell lines are extensively utilized in a wide range of biological analyses. Currently, cell cultures are utilized for the research of various biological processes [73]. However, the importance of appropriate cell culture method selection in cancer research represents the key condition for a better understanding of biological processes observed [74].

### 2.1. In Vitro Experimental Models Utilization

#### 2.1.1. Monolayer Cell Culturing—2D Cell Experimental Model

Original cell phenotype maintenance in cells utilized for laboratory experiments represents the key effort in the field of cellular biology analyses [75,76]. Nowadays, the utilization of 3D cell models is becoming more popular. Nevertheless, the 2D cell model is dominantly used in a huge amount of molecular analyses [77]. Deficiencies and limitations of this experimental model are relatively well described, but we must define some possible limitations of this model concerning specific biological mechanisms focused on a problem complexity consideration. On the other hand, it is important to mention that the investigation in the field of PDT in relation to a tumor angiogenic mechanism was performed primarily with animal experimental models (see Table 1 and Table 2). However, the utilization of less relevant 2D cell models also occurred [28,60,61,62,63,64,65] (see Table 3).

The surviving cell fraction represents one of the most serious problems after PDT observation. Chen assumed that PDT with hypericin (HY) could probably induce immediate cell death only in 30% of fibrosarcoma tumor mass [50]. On the contrary, Kamuhabwa et al. [78] documented only a small fraction of surviving cells, estimated from 2–5%, in a bladder carcinoma with the same PS utilization. Nevertheless, the existence of this fraction was associated with tumor regrowth mechanism induction [78] and angiogenic factors upregulation [15].

The observed surviving cell fraction after PDT could be connected with fundamental components for PDT realization, which means light, oxygen, and PS presentation in tumor tissue [79]. In this context, the toxicity of the PS prevalently depends on light of the appropriate wavelength and oxygen presence. In a subsequent cascade, the production of reactive oxygen species (ROS) leading to destruction of targeted cells or tissues is utilized [80,81]. It is obvious that the tumor’s distribution of the mentioned components does not have homogenous properties, and is directly associated with the spatial characteristics of solid tumors. Tissue light penetration is limited by light wavelength characteristics, whereas in some range of radiation, the longer wavelengths can penetrate deeper than shorter wavelengths. Specifically, the red light with higher wavelength characteristics has maximum penetration depth, approximated to 5 mm [82], and only tumor cells within 70–150 µm of tumor vasculature [83,84] can consume oxygen. Oxygen insufficiency, known as hypoxia, is a common feature of solid tumors. The development of hypoxic regions is associated with lowered therapeutic response and tumor progression. The cells inhabiting hypoxic regions of tumors have a higher resistance to chemotherapy and radiotherapy, in comparison to the cells localized in normoxic tumor regions. Since PDT directly depends on oxygen presence, tumor hypoxia represents one of the most restricting aspects of PDT effectiveness [81]. Moreover, tumor vascular permeability is very heterogeneous and could decrease the accessibility of PS in targeted tumor cells [85]. In this context, Khan et al. [81] recently summarized the effect of the enhanced permeability and retention (EPR), defined as a tumor’s ability to accumulate particles in the size range of 380–720 nm. The authors also concluded that oxygen containing microbubbles and nanobubbles (MNBs) may enhance the therapeutic efficiency of PDT.

The spatial characteristics of the 2D cell model represent a major restriction for its utilization, as this model could not consider any of the above-mentioned factors. In 2D cell models, light and oxygen have relatively homogenous distribution through a total cell population. Moreover, the impossibility of mimicking the tumor vasculature effect on PS permeability into the tumor leads to its inadequate accessibility associated with a significantly higher cell accumulation. Finally, comparative analyses in relation to PDT showed that the same treatment regimens cause significant differences in cytotoxic effects. Within the given frame of references, the cells cultivated in the 2D cell model exhibit higher sensitivity to PDT, and therefore, the rate of surviving cells fraction in equal treatment conditions is minimalized. As was mentioned, a PS accumulation level is associated with a total cytotoxic effect, subsequently affecting angiogenic factor expression. Therefore, the consideration of the third dimension seems to be a very convenient approach for mimicking spatial tumor characteristics [14,86,87,88,89,90]. It is important to state that prevalently decreased proangiogenic growth factors, like PD-ECGF and FGF-2 protein expression after PDT with hypericin (HY-PDT) in HCT 116 cells [14], VEGF after PDT with aminolevulinic acid (ALA-PDT) in SW620 [61,62], and PDT with meta-tetra(hydroxyphenyl)chlorin (mTHPC-PDT) in VB6 and H376 cells [64], were observed if a 2D cell experimental model was utilized. This could be associated with the above-mentioned aspects of sufficient PS penetration in the whole targeted cell population [14]. However, there are also opposite findings documented when prostaglandin COX-2 upregulation as another type of angiogenic factor after PDT with HY [59] photofrin (PT) [28,29] and piropheophorbide-a methyl ester (PPME) [68] were detected with the utilization of 2D cell models. This evident discrepancy might point to the fact that only spatial characteristics consideration is not a sufficient condition guaranteeing experimental model relevancy in a tumor angiogenesis research, and that there must be some other important aspects to be considered. Angiogenic factors represent a wide spectrum of molecules potentiating vascularity creation. However, their synthesis has been described in a wide range of malignant and non-malignant cell types. Some angiogenic factors, such as growth factors, are expressed in a broad variety of cell types [91,92,93]. Synthesis of other angiogenic factors, e.g., prostaglandins like COX-2, are more specific for cancer cells in tumors [94,95,96,97,98,99]. The expression of other angiogenesis-promoting factors, like MMP-2 or MMP-9 [43,100] has been prevalently detected in tumor stroma. As it was mentioned above, the PDT effect on cells cultivated in the 2D cell model is more frequently associated with a lowering of angiogenic growth factor protein expression in cancer cells [14,61,62,63,64]. On the contrary, with in vivo conditions, some drastic lowering of growth factors synthesis after PDT was not observed, and their inhibition was prevalently associated with the utilization of some antiangiogenic agents (see Table 1). Moreover, the effect of the mentioned therapy in vivo is predominantly associated with a progressive increase in angiogenic factor expression. Furthermore, the possibility for long-lasting analyses with in vivo experimental model utilization, in comparison to the 2D cell model, can reflect the secretion potential of the total tumor cell population. In the context of this information, it seems to be evident that a monoculture 2D cell model has very limited application in analyzing angiogenic factor expression after PDT. Its utilization could be potentially considered for some preliminary analyses in relation to factors expressed more specifically in a particular cell type, as it was detected in COX-2 [94,95,96,97,98,99], but it is evident that a majority of angiogenic factors, with less specific cell expression characteristics, could not be analyzed with the utilization of a monoculture 2D cell model, as this model does not have potential to offer a holistic view of the angiogenic factor expression, reflecting the reaction of whole tumor mass affected.

#### 2.1.2. Spheroids: 3D Cell Experimental Model and Preservation of Tissue-Like Characteristics

The history of 3D cell cultures started in the 1970s [101]. Until now, a lot of methods for spheroid creation have been developed, but they are not going to be discussed, as it is not the goal of this article, and there is still growing evidence of review papers dealing with this topic [102,103,104].

In the context of the above-mentioned information, there is no doubt that the presence of spatial characteristics in the experimental model utilized for PDT concerning tumor angiogenesis mechanism research is fundamentally needed. The geometry of the experimental model is sufficient enough for alternative splicing angiogenic factor alterations, like vascular endothelial growth factor (VEGF) in malignant cells [105]. Nonetheless, only the presence of spatial characteristics in experimental models utilized for PDT effects analyzed in connection with tumor angiogenic mechanism is still insufficient.

Concerning novel methods and experimental model utilization in cancer research, we must abandon a simplistic description of solid tumors as complex structures of genetically modified cells with high clonogenic capabilities [106,107].

The last decade is characteristic of the growing interest in tumor environment study, as there have been many potential antitumor targets identified [108]. Finally, it is very important to emphasize that non-malignant cell fractions gain some specific characteristics if they are placed in a tumor environment, known as a tumor-associated process. Currently, there is a huge scale of tumor-associated cell types, such as cancer-associated fibroblasts, endothelial cells (ECs), pericytes, adipocytes, and other types of immune cells [109,110,111,112]. With the objective of the interaction of tumor stroma with cancer cells rate in multicultural in vitro 3D cell models, Del Bufalo et al. [21] analyzed a novel 3D modeling PEG-fibrin hydrogel system, and described higher similarity with in vivo conditions in relation to morphological features and treatment response. Besides that, there was a more aggressive phenotype inducted, and higher proliferative and metastatic status observed, if cancer cells were co-cultivated with fibroblasts and ECs. Moreover, it was shown that fibroblasts alone, or in combination with ECs, stimulate tumor growth and metastatic potential [21].

The above-mentioned facts are important not only for our better understanding of tumor stroma effect on tumor phenotype characteristics, but they also show that, in 3D cell models, these interactions are developed [21,113,114]. Furthermore, Amann showed that in multicultural spheroids composited of cancer cells, fibroblasts, and ECs, alpha smooth muscle cell (ASMA) expression is induced in all cell types [22]. Its presence in fibroblasts is associated with tumor-associated phenotype gains, while in cancer cells, it is defined as epithelial to mesenchymal transition (EMT) [115,116]. Contrarily, ASMA was not expressed if the cells were cultivated in monocultural spheroids [22].

Besides that, it was shown that the tumor angiogenic mechanism is directly modulated by the presence of the tumor microenvironment. This mechanism is associated with complexity and reciprocal interaction of multiple tumors, creating cell types significantly influencing the angiogenic expression profile of tumors, as was proven by multicultural spheroids utilization. Data showed that tumor-associated fibroblasts were not just the major producer of mentioned growth factors. However, if they were co-cultivated with cancer cells, a significant increase of VEGF and FGF-2 secretion by analyzed spheroids was observed. The mentioned information means that a non-malignant section of tumor mass has a key impact on the level of expression of angiogenic factors in malignant cells. Consequently, a significant upregulation of the expression of angiogenic factors in malignant cells was induced [22]. Nevertheless, the elevated level of expression of growth factors by tumor-associated fibroblasts is only one aspect of the problem [117,118,119]. It was also demonstrated that biomechanical activity of tumor-associated fibroblasts could regulate vascular growth, and might potentiate the formation of blood vessels in the tumor microenvironment. Mechanotransductive pathways like Rho/Rho-Associated Coiled-Coil Forming Kinase Pathway (ROCK), The Hippo/yes-associated protein signaling pathway (YAP), and Snail1 were also shown to be associated with vessel growth [120]. All the mentioned information gained from 3D cell models highlights the fact that a tumor angiogenesis process is very complex, and if only one aspect is taken into consideration, the results do not have to be favorable, as was observed with antiangiogenic therapies targeted on a particular growth factor [121].

Although the fact that there is a massive effort to develop novel 3D in vitro experimental models and that novel information proved its possible utilization in a huge range of cancer types, its optimization has occurred mainly with lung adenocarcinoma [22]. Based on the information mentioned, we assume that the optimization and preparation of that model for other types of cancer are also necessary. Finally, based on findings gained from 3D multicultural experimental models [21,113,114,120] in connection to the tumor angiogenesis research associated with PDT, we have to re-evaluate the utilization of not only the 2D monocultural cell models, but also all of the models, that cannot reflect the interaction of tumors microenvironment [122,123].

## 3. Chorioallantoic Membrane of Avian Embryo (CAM)

The CAM of an avian embryo is a structurally simple, highly vascularized extraembryonic membrane. Except for a respiration function, allantois also serves as a reservoir of waste products of the embryo. The CAM also functionally participates in sodium and chloride transport from the allantois cavity and calcium transports from the egg shell to start the bone mineralization [124]. For research purposes, CAM vasculature is most interesting. Besides the vascular system, the CAM also has a fully developed lymphatic system, very similar to mammalian lymphatics [125].

Structurally, the CAM consists of two epithelial sheets. The upper epithelium is of ectodermal origin, while the stroma and the lower epithelium are of mesodermal and endodermal origin, respectively. Blood and lymphatic vasculature are located in a stromal part, so every compound delivered on the CAM surface has to pass across the surface epithelial layer, and reach vessels in the stroma [126].

Moreover, the CAM is simple to handle, its utilization is low-cost, and ethics committee approval is free to obtain, as an avian embryo is not considered as a living animal until day 17 of its development in most countries [127]. The CAM could be also utilized for transplantation analyses without any immune response, as it is immunodeficient. The immune system deficiency enables the utilization of avian embryos for the cultivation of a wide range of biological materials, such as tumor xenografts [128] and 3D cell models [129]. Furthermore, the latest data shows that a cell suspension of the appropriate density could be topically applied on the CAM surface for the creation of micro-tumors [14]. Moreover, the CAM maintains many cancer characteristics, such as growth, invasiveness, and angiogenic potential. Furthermore, the remodeling of the tumor microenvironment is also possible. In addition, genetic analyses have shown that the chicken genome has approximately the same number of genes as a human genome, with a high level of sequence conservation [127].

However, quick rearrangement of the vascular system limits the precision of the detection of novel vessels [130]. Moreover, the CAM is also very sensitive to many environmental factors, such as oxygen tension, pH, etc. [131]. However, possibly the most obligatory limitation in CAM utilization is its low compatibility with a wide range of reagents, such as antibodies, cytokines, and primers [127].

The extensive vascular potential of the CAM makes it possible to analyze the effect of a wide spectrum of substances on an angiogenic mechanism. The analyzed substances are topically applied on the CAM surface [132], and the effect can be observed 72 h after its application. The angiogenic properties of analyzed materials are displayed in vascular density changes in the surrounding environment of the analyzed material. A higher vascular density is associated with proangiogenic potential, while a lower vascular density is observed if the antiangiogenic potential is proven [127].

The CAM vasculature provides extensive possibilities for PDT analyses. PDT induces reactive oxygen species production that subsequently activates a cascade of chemical and physiological processes, initiating the creation of occlusions on the CAM [133]. The PS could be applied intraperitoneally [134], topically [135], or intravascularly [133,134,136,137,138,139]. As many PSs are hydrophobic, quickly accessible vascularity enables us to analyze novel carriers, such as liposomes [30,139], solvents, e.g., N-methyl pyrrolidone [32], polyether compounds, such as polyethylene glycol [34], or liposomal nanoparticles [139]. The effects of PDT on normal vasculature of avian embryo with the utilization of different PSs, such as VT [140,141], BPD-MA, and mTHPP [142] or HY [34], have also been analyzed. However, in connection with tumor angiogenesis research, we assume that a healthy vascular system of an avian embryo individually could not represent a convenient experimental model, as it does not have the potential to mimic the pathological changes observed in tumor vasculature. Regardless, the latest information has shown that micro-tumors created on aviary embryos are structurally interconnected with the CAM. They have a proliferative active status and could be utilized for tumor angiogenesis research associated with molecular analyses focused on the angiogenic mechanism after PDT. Besides the fact that spatial features directly affect the total HY-PDT cytotoxic effect, some differences in angiogenic factor expression between the 2D cell model and experimental micro-tumors were also observed. While a gene expression upregulation was predominantly detected in both analyzed experimental models, at the protein level, no effect on experimental micro-tumors was observed. Contrarily, in the 2D cell model, downregulation of PD-ECGF and 24-kDa isoform of FGF-2 in HCT 116 cells was also observed. The mentioned effects were most likely connected with different accumulation status of HY in targeted intracellular organelles between analyzed experimental models. As the HY also accumulates in a significant manner in protein synthesizing cellular organelles, their massive destruction could represent a dominant reason for the observed discrepancy between gene and protein expression in 2D cell models affected by HY-PDT. Moreover, the observed downregulation of protein expression could be only temporary, and long term analyses have to be realized. On the other hand, a micro-tumor’s utilization does not make a long-term effect after PDT observation possible [14]. As the long-term effect analysis in relation to angiogenesis after PDT incidence represents a very important part of the research, supplementary analyses on other experimental in vivo models are needed [15,19,20,23,37,38,39,40,41,48].

## 4. In Vivo Mammalian Experimental Models

As mentioned above, the majority of experimental analyses focused on PDT concerning tumor angiogenesis were performed with in vivo mice or rat experimental model utilization. In this context, the fundamental requirements, such as the multicultural character of experimental tumors, the possibility of vascular application of PS, and the long-term effect observation, could be considered as the major advantages of these experimental models for tumor angiogenesis research after PDT. The therapeutic effect of PDT with a wide range of PSs utilized was associated mainly with the induction of the tumor angiogenesis mechanism (see Table 1). Ferrario et al. [42], in some initial hypotheses, proclaimed that VEGF upregulation in PT-PDT treated tumors is predominantly induced because of hypoxia development after treatment. Currently, we know that hypoxia represents one of the key mechanisms triggering angiogenesis by the HIF-1α factor, which is involved in VEGF transcription [143], which is in correlation with Ferrario’s observations [42]. The importance of hypoxia in VEGF stimulation in PDT-treated tumors with other PSs, such as HY, was also confirmed [36]. On the other hand, the later analyses showed that angiogenesis is not only indirectly supported by hypoxia, but also that PDT with the utilization of different PSs could stimulate the expression of proangiogenic factors in cancer cells. The results from analyses of growth factors, such as VEGF after ALA-PDT [61,62] or mTHPC-PDT [64] or FGF after ALA-PDT [63], obtained from 2D cell experimental models did not generally point out the proangiogenic PDT potential as a consequence of protein expression not increasing. Contrarily, some gene expression analyses showed a significant VEGF upregulation after HY-PDT [14] and mTHPC-PDT, where the upregulated extracellular secretion level of VEGF was also detected [65].

As a possible solution for the expression of angiogenic factor lowering in cancer cells, PDT targeted mainly on tumor vasculature with HY [48,49,50] or BPD-MA utilization was analyzed [55,56]. Besides the fact that there were totally cured experimental animals after HY-PDT described [48,51], the extensive side effects associated with massive destruction of non-malignant tissues were detected [48,49,50,51,55]. Based on that, it is possible to assume that the side effects associated with vascular-targeted PDT could represent a major limiting factor for its future utilization in cancer treatment. Moreover, there are many pieces of evidence pointing out that non-malignant cells or tissues affected by PDT could also represent a rich source of angiogenic factors.

In this context, Bhuvaneswari et al. [15] observed a modest, but statistically significant, mouse VEGF increase in tumor 48–72 h after PDT with HY utilization on the murine model of human nasopharyngeal carcinoma. Similar results were described, concerning Kaposi’s sarcoma after PT-PDT [16]. Interestingly, Gallagher-Colombo [17] noted approximately ten times higher VEGF upregulation already by host mice cells 24 h after VT-PDT application on non-small cell lung carcinoma. Moreover, Jiang detected upregulated VEGF in PDT-affected normal brain tissue, even six weeks after PT-PDT. Furthermore, a neovascular expansion four weeks after treatment in affected brain tissue was observed [18]. As a possible solution for the elimination of massive vascular expansion, Zhang et al. [19] subsequently pointed to the application of lower PDT doses (2 J/cm^2^). Nevertheless, significantly increased ECs proliferation and VEGF upregulation were also detected, in comparison to the untreated contralateral brain region. Subsequently, immunohistochemical analyses with PT-PDT utilization showed that the brain adjacent to tumor tissue also represents a rich source of VEGF, and neither vascular endothelial growth factor receptor-1 (VEGFR-1), MF1, nor vascular endothelial growth factor receptor 2 (VEGFR-2), DC101 antibody application could decrease VEGF expression to non-treated control levels [20]. Similar findings with PT-PDT utilization were observed for fibrosarcoma with NS-398 COX-2 inhibitor [27], and in vitro after p38 mitogen-activated protein kinase (MAPK), SB203580, and SB202190 inhibitor application [29].

In addition, combination treatment analyses with ALA-PDT were also performed on Walker carcinosarcoma with chitosan [24] and bladder carcinoma with deferoxamine [25] utilization. Interestingly, the authors of both mentioned studies pointed to a higher PS accumulation when a combined treatment regime was applied, in what could be considered as a very important benefit in connection to fundamental components of PDT. Moreover, Inoue noted a significantly reduced intratumoral vasculature 24 h after PDT [25]. Nevertheless, after combined treatment with chitosan utilization, there were not any alterations in MMP-2 expression observed. On the other hand, only in comparison to the ALA-PDT experimental group, an already decreased level of pro-enzyme form of MMP-2 1 h after a combined treatment application was described [24].

Humanized anti-VEGF monoclonal antibody bevacizumab, in combination with mTHPC-PDT, decreased VEGF expression in correlation to the mTHPC-PDT group, but on the contrary to chitosan and deferoxamine utilization in ALA-PDT [24,25] the reduction mTHPC intratumoral accumulation was also detected [26].

A combined therapy utilized in relation to HY-PDT seems to be very promising for angiogenesis inhibition. In comparison to non-treated tumors, a significant downregulation of VEGF expression on mRNA [23] and protein [15,23] levels was observed even 24 days after treatment application. However, the mentioned analyses were performed only concerning nasopharyngeal carcinoma [15,23].

The above-mentioned information has shown that PDT, with the huge scale of PS utilized, induces an angiogenic mechanism in a wide range of tumor types (see Table 1). In addition, non-malignant tissues also represent very important producers of angiogenesis-promoting molecules, as was predominantly showed in PT-PDT on the brain adjacent to the tumor [20] or normal brain tissue [18]. One of the possible solutions could be the utilization of multiple angiogenesis inhibitors targeted on host and tumor cells, as it was with VT-PDT and mouse anti-VEGF antibody and Bevacizumab utilization [17]. Contrarily for HY utilization, there are some pieces of evidence describing very specific accumulation in tumor mass [48], therefore a lowered expression of angiogenic factors after HY-PDT, in combination with antiangiogenic therapy utilization even 24 days after treatment, could be also associated with this fact. Moreover, it could be supposed that HY was predominantly accumulated in cancer cells of a tumor. Regardless, the additional analyses must be performed to verify this assumption.

Concerning that, a higher level of cancer cell-specific accumulation of PS is fundamental because non-malignant cells could potentiate tumor growth by angiogenic factor expression after PDT [15,16,17,18,19,20]. Nonetheless, as the data showed, the application of angiogenesis-inhibiting factors in PDT seems to be necessary. Based on that, both angiogenic factor inhibition and the maintenance of PS accumulation in cancer cells are essential for a sufficient therapeutic effect. Simultaneously, a development and analysis of convenient PS carriers enhancing PS accumulation could significantly improve the therapeutic effect of PDT, mainly concerning angiogenic mechanism.

## 5. Conclusions

Induction of angiogenic mechanism after PDT represents a serious problem associated with a tumor regrowth development in affected tumors. Besides that, the analyses have shown that there are many factors regulating tumor angiogenesis, and the tumor stroma represents the key regulator of this mechanism in many aspects. The tumor stroma fundamentally affects the behavior and phenotypical characteristics of malignant and non-malignant cells. Additionally, there is evidence that angiogenesis stimulation in cancer is not only associated with malignant cells, but also that the non-malignant cell fraction represents an important source of angiogenesis stimulating factors. Moreover, PDT could also induce angiogenic mechanism in these normal cells. Therefore, the preservation of tumor microenvironment conditions and multicultural characteristics in experimental models represents an essential requirement for PDT research about angiogenesis. Simultaneously, the improvement of PS accumulation in targeted tumor cells associated with novel carriers and angiogenesis inhibitors development represents a cardinal challenge in the field of angiogenesis research about PDT.

## Figures and Tables

**Table 1:** In vivo experimental models utilized for photodynamic therapy (PDT) in relation to angiogenesis, focused on analyses of angiogenic factors.

Experimental Model; Type of Tumor (Cell Line)	PS	PS Administration	PS Doses and Accumulation Time	Light Dose; Fluence Rate	Effect on Angiogenesis	References
2D in vitro, male BALB/c nude mice; human nasopharyngeal carcinoma (CNE2)	HY	i.v.	2 mg/kg; 2 h	42.4 J/cm^2^; 56 mW/cm^2^	* PDGF-B detected in hypoxic conditions in vitro and non-detected in vivo (24 h) * ↓ COX-2 (6–24 h) and HIF-1α (24 h) * ↓ COX-2, HIF-1α, VEGF in combined treatment with Celebrex (24 days)	[23]
Balb/c nude mice; human nasopharyngeal carcinoma (HK1)	HY	i.v.	2 or 5 mg/kg; 1 h or 6 h	30 J/cm^2^; 25 mW/cm^2^	* after 6 h accumulation time ↓ serum VEGF in comparison to 1 h accumulation (24 h)	[36]
male BALB/c athimic (nu+/nu+) mice; human nasopharyngeal carcinoma (CNE2)	HY	i.v.	2 mg/kg; 2 h	47.7 J/cm^2^; 6 mW/cm^2^	* ↑ VEGF, COX-2, HIF-1α, FGF-2, PDGF-β (24 h)	[37]
Balb/c nude mice; human nasopharyngeal carcinoma (HK1)	HY	i.v.	5 mg/kg; 6 h	120 J/cm^2^; 50 mW/cm^2^	* ↓ human VEGF in serum and tumor (24 h) and ↑ (72 h)	[15]
male Balb/c nude mice; human bladder carcinoma (MGH)	HY	i.v.	5 mg/kg^−1^; 6 h	50–150 J cm^−2^; 42–125 mW cm^−2^	↓ FGF-2, EGF, PlGF, TIMP1,2, VEGF, IL6 and IL8 in combined treatment with Avastin (30 days)	[38]
Balb/c nude mice; human bladder carcinoma (MGH)	HY	i.v.	5 mg/kg; 0.5 h (short drug-light interval), 6 h (long drug light interval)	120 J/cm^2^; 100 mW/cm^2^	* ↑ VEGF after long drug light interval and ↑ FGF-2 after short drug light interval protein expression (24 h)	[39]
2D, 3D, quail CAM; human colon cancer (HT-29, HCT 116), mice colon cancer (CT26.WT)	HY	t.a.	25 nM–1 µM; 16 h	3.15 J∙cm^2^; ---	* differences of HY accumulation between 2D and 3D cell model (16 h) * proved HY penetration into the micro-tumors * mainly ↑ of growth factors gene expression in 2D and micro tumors (24 h) * ↓ PD-ECGF, FGF-2 protein expression in 2D cell model (24 h) * in micro-tumors unaltered protein expression of growth factors (24 h)	[14]
CC57 Bl/6 mice; mouse Lewis lung carcinoma (LLC and LLC/R9, respectively)	Hem	i.p.	Dose of Hem in the conjugate with antiVEGF: 0.04–0.05 mg per animal; 24 h	initial capacity: 25 mW; radiation dose: 50 W s/cm^2^	* ↑ tumor growth inhibition and survival of experimental animals if HEM was conjugated with antiVEGF antibodies	[40]
C57B1/6 mice; mouse Lewis lung carcinoma (3LL)	ALA	p.o.	500 mg/kg; ---	---; 150 mW/cm^2^	*↓ VEGF in serum (10–11 days)	[41]
male Wistar rats; grafted with small fragment of Walker tumor in the right thigh	ALA	i.p.	250 mg/kg; 3 h	50 J/cm^2^; 25 W	* ↓ activity of MMP-2 in combined treatment with chitosan (1 h and 24 h)	[24]
female BALB/c athymic (nu+/nu+) mice; human colon carcinoma (HT-29)	mTHPC	i.v.	0.3 mg/kg; 24 h	10 J/cm^2^; 100 mW/cm^2^	* ↓ VEGF and microvessel density, in combined treatment with bevacizumab (1 week)	[26]
2D, female C3H/HeJ mice; mouse mammary carcinoma (BA)	PT	i.v.	5 mg/kg; 24 h	200 J/cm^2^; 75 mW/cm^2^	* ↑ HIF-1α and VEGF in tumors (24 h) * ↑ VEGF in vitro only in chemically induced hypoxia (CoCl_2_) conditions (2–24 h)	[42]
2D, C3H/HeJ mice; mouse fibrosarcoma (RIF), mouse mammary carcinoma (BA), mouse Lewis lung carcinoma (LLC)	PT and NPe6	i.v.	5 mg/kg; 24 h	200 J/cm^2^ and 300 J/cm^2^; 75 mW/cm^2^	* ↑ COX-2 in RIF cells after PT-PDT (1.5 h and 3 h) * ↑ COX-2 after NPe6-PDT (24 h) and after PT-PDT (24 h -192 h) * ↑ VEGF in RIF tumors (24 h) * ↓ VEGF in RIF tumors after PT-PDT with NS-398 utilization	[27]
Normal rat brain	PT	i.p.	12.5 mg/kg; 24 h	140 J/cm^2^; 100 mW/cm^2^	* ↑ VEGF immunoreactivity (1–6 weeks) and vessel branching (3–6 weeks)	[18]
female C3H/HeJ mice; mouse mammary carcinoma (BA), mouse brain ECs, mouse macrophages (RAW 264.7)	PT	i.v.	in vitro: 25 µg/mL; 16 h; in vivo: 5 mg/kg; 24 h	in vitro: ---; 0.35 mW/cm^2^; in vivo: 0 to 200 J/cm^2^; 75 mW/cm^2^	* ↑ MMP-9 expression (24 h) and gelatinase activity in BA tumors (24–48 h) * BA cells in vitro secreted only detectable levels of MMP-2 * ↑ pro-MMP-2 and pro- and activated MMP-9 in medium of ECs (24 h) * ↓ MMP in macrophages in vitro (24 h)	[43]
Athymic mice; Normal mice brain	PT	i.p.	2 mg/kg; 24 h	2 J/cm^2^ or 4 J/cm^2^; ---	* ↑ ECs proliferation (1–2 weeks) * ↑ VEGF immunoreactivity (4 J/cm^2^; 1 week)	[19]
C3H/HeJ mice; mouse mammary carcinoma (BA)	PT	i.v.	5 mg/kg; 24 h	0–200 J/cm^2^; 75 mW/cm^2^	* ↓ of VEGF and PGE_2_ in combined treatment with celecoxib or NS-398 (24 h)	[44]
athymic nude mice; rat gliosarcoma (9L)	PT	i.p.	2 mg/kg; 24 h	40, 80 or 120 J/cm^2^; ---	* unaltered VEGF in tumors (1 week) * ↑ VEGF in brain adjacent to tumor (120 J/cm^2^; 1 week)	[45]
athymic nude mice; human glioblastoma (U87)	PT	i.p.	2 mg/kg^−1^; 24 h	80 J/cm^-2^; ---	* ↑ of VEGF and von Willebrand factor (vWF) positive vessels (2 weeks) * ↓ VEGF and vWF positive vessels if VEGFRs antibodies were utilized (2 weeks)	[20]
male severe combined immunodeficient (SCID) mice; human prostate carcinoma cells (LNCaP)	BPD-MA	i.v.	0.25 mg/kg; 1 h	100 J/cm^2^; ---	↑ VEGF (24 h)	[46]
male severe combined immunodeficient mice; human prostate carcinoma (LNCaP)	BPD-MA	i.v.	in vitro: 140 nM/l; 1 h; in vivo: 0.25 mg/kg; 1 h	in vitro: 690 nm; in vivo: 50 J/cm^2^; 100 mW/cm^2^	↑ VEGF in cancer cells and tumors (24 h)	[47]
nude mice; human lung carcinoma (H460)	VT	i.v.	1 mg/kg; 3 h	---; 75 mW/cm^2^	↓ human and murine VEGF in combined treatment with anti-mouse antibody and bevacizumab (24 h)	[17]

i.v., intravenous; t.a., topical application; i.p., intraperitoneal; p.o, *per os*; *, particular information related to angiogenesis; ---, the parameter was not provided by the authors.

**Table 2:** In vivo experimental models utilized for PDT in relation to angiogenesis.

Experimental Model; Type of Tumor (Cell Line)	PS	Ps Administration	Ps Doses and Accumulation Time	Light Dose; Fluence Rate	Effect on Angiogenesis	References
female C3H/Km mice; mouse fibrosarcoma (RIF-1)	HY	i.v.	5 mg/kg; 0.5 h, 6 h, 24 h	120 J/cm^2^; 100 mW/cm^2^	* 100% cured animals after 0.5 h accumulation time, but massive skin necrosis was detected *delayed tumor growth after 6 h accumulation time	[48]
female DBA/2 mice; mouse lymphoma (P388)	HY	i.v.	1 mg/kg; 1 h, 5 or 20 mg/kg; 24 h	120 J/cm^2^; 100 mW/cm^2^	*the best therapeutic effect was observed if 0.5 h accumulation time was utilized, but massive skin necrosis was detected	[49]
female C3H/Km mice; mouse fibrosarcoma (RIF-1)	HY	i.v.	5 mg/kg; 0.5 h, 6 h, 24 h	120 J/cm^2^; 100 mW/cm^2^	* 6 h accumulation time induced 30% direct cell death of tumor cells * after 0.5 h accumulation time no direct cell death was observed, but skin necrosis was detected	[50]
female Balb/c mice; mouse colon carcinoma (CT26)	HY	i.v.	low-power PDT: 2.5 mg/mL; 0.5 h, high power PDT: 10 mg/mL; 1 or 4 h	low-power PDT: 14 J/cm^2^; 27 mW/cm^2^; high power PDT: 60 J/cm^2^; 50 mW/cm^2^	* after low-power PDT all tumors completely disappeared, and necrosis formation started * after high power PDT with 4 h incubation time tumor growth reduction was observed * in high power PDT after 1 h incubation time mice died	[51]
quail CAM	HY	t.a.	2 µg/g; 1 h or 5 h	16.8 J/cm^2^; 140 mW/cm^2^	* massive vasculature damage after 1 h and 5 h incubation time	[34]
CC57 Bl/6 mice; mouse Lewis lung carcinoma (LLC and LLC/R9, respectively)	HT	i.p.	HT in the conjugate with antiVEGF: 0.04–0.05 mg per animal; 24 h	initial capacity: 25 mW; radiation dose: 50 W s/cm^2^	* ↑ tumor growth inhibition and survival of experimental animals if HT was conjugated with antiVEGF antibodies	[40]
male athymic BALB/cA Jc 1-nu nude mice; human bladder carcinoma (253 J B-V)	ALA	i.p.	50 mg/kg; 1.5 h	100 J/cm^2^; 100 mW/cm^2^	* ↓ CD31 positive vessels in combined treatment with deferoxamine	[25]
female Wistar rats; chemically Induced premalignant lesion on the tongue	ALA	cream composed of 5% 5-ALA	---; 2 h	90 J/cm2; 40 mW	* ↓ CD34 positive vessels in comparison to other experimental groups * in comparison to non-treated control group the number of CD34 positive vessels ↑	[52]
athymic nude mice; Ewing’s sarcoma (A673)	PT	i.v.	10 mg/kg; 24 h	150 J/cm^2^; 250 mW/cm^2^	blood flow ↓ and progressive disruption of blood vessels endothelial layer in affected tumors	[53]
athymic nude mice; human glioblastoma (U87)	PT	---	10 mg/kg; 48 h	10 Gy radiation	* unchanged microvessel density after PDT	[54]
Balb/c male mice; mouse fibrosarcoma (Meth A)	BPD-MA	i.v.	0.5–2 mg/kg; 15 min-3 h	150 J/cm^2^; 0.25 W	* ↓ tumor growth observed in both accumulation times * after 0.5 h accumulation time body weight lost was observed and 30% of animals died * if 0.5 mg/mL of photosensitizer was applied better therapeutic effect was observed	[55]
Balb/c male mice; mouse fibrosarcoma (Meth A)	BPD-MA	i.v.	0.25 mg/kg; 15 min	150 J/cm^2^; 0.25 W	* remarkable vasculature damage after PDT with utilization of polycation liposomes	[56]
female normal BALB/c mice; mouse breast carcinoma (4T1)	BPD-MA	i.v.	1 mg/kg; 24 h	120 J/cm^2^; ---	* ↓ microvessel density in PDT alone or in combined treatment with adriamycin	[57]
male BALB/c mice; mouse fibrosarcoma (Meth A), human ECs (ECV304)	VT	i.v.	0.25 mg/kg; 15 min or 3 h	150 J/cm^2^; 0.25 W	* vasculature photodamage was observed after 15 min accumulation time	Ichikawa [58]

i.v., intravenous; t.a., topical application; i.p., intraperitoneal; *, particular information related to angiogenesis; ---, the parameter was not provided by the authors.

**Table 3:** In vitro experimental models utilized for PDT in relation to angiogenesis.

Type of Tumor (Cell Line)	PS	PS Doses and Administration	Light Dose/Fluence Rate	Effect on Angiogenesis	References
human cervical adenocarcinoma (HeLa), human urinary bladder carcinoma (T24)	HY	125 or 150 nM; 16 h	4 J/cm^2^; 4.5 mW/cm^2^	* ↑ COX-2 (3–24 h)	[59]
human ECs (HUVEC), human likely glioblastoma (U-87 MG), human glioblastoma (U-373 MG)	HY	5 × 10^−9^–5 × 10^−7^ mol/L; 3 h	5 J/cm^2^; 3.22 mW/cm^2^	* ↓ MMP-9 in HUVEC and MMP-2 in HUVEC and U-87 cells * tubulogenesis inhibition by HUVEC cells (24 h)	[60]
human colorectal adenocarcinoma (SW480, SW620)	ALA	1000 mM; 4 h	10 J/cm^2^; 0.3–1.5 mW/cm^2^	* ↓ FGF in SW620 cells in normoxic or simulated hypoxic (by CoCl_2_) conditions and ↓ VEGF in SW620 cells (24 h)	[61,62,63]
human oral squamous cell carcinoma (H376, VB6 and UP)	mTHPC	0.25–4 µg/mL; 24 h	0.25–4 J/cm^2^; 25 mW/cm^2^	* ↓ MMP-9 and MMP-13 in H376 cells * ↓ MMP-2 and ↑ MMP-13 in VB6 cells * ↓ MMP-2 and VEGF in H376 (24–48 h)	[64]
human epidermoid carcinoma (A431)	mTHPC	0.1 µg/mL^−1^; 18 h	---; 1.6 mW cm^-2^	* ↑ VEGF and IL1A (4–24 h)	[65]
C-26 cells	PT	10 µg/mL; 24 h	4.5 kJ/m^2^; ---	* ↑ COX-2 (2–24 h)	[28]
human glioblastoma (U87, U-118 MG)	PT	10-50 mg/mL; 4 h	1 J/cm^2^; ---	* ↓ invasion and angiogenesis network potential after PDT (72 h) * ↓ VEGF, FGF-2, EGFR, MMP-2 and MMP-9 and also Akt and NF-κB (24 h)	[66]
human ECs (HUVEC)	VT	1–2 µM; 90 min	36 J/cm^2^; 0–300 mW/cm^2^	* PDT with fVII factor killed almost 90% of VEGF-stimulated HUVECs but had no effect on unstimulated HUVECs.	[67]
human urinary bladder carcinoma (T24), human cervical adenocarcinoma (HeLa)	PPME	5 µM; 3 h	3.2 J/cm^2^; 160 W/m^2^	* ↑ COX-2 in both cell lines 3 h after PDT * 9–12 h after treatment ↓ COX-2 in HeLa cells to basic level	[68]

*, define concrete finding related to angiogenesis; ---, the parameter was not provided by the authors.
